# Effect of community based interventions on childhood diarrhea and pneumonia: uptake of treatment modalities and impact on mortality

**DOI:** 10.1186/1471-2458-13-S3-S29

**Published:** 2013-09-17

**Authors:** Jai K Das, Zohra S Lassi, Rehana A Salam, Zulfiqar A Bhutta

**Affiliations:** 1 Division of Women & Child Health, The Aga Khan University, Karachi, Pakistan; 2 Global Child Health and Policy, Centre for Global Child Health, The Hospital for Sick Children, Toronto, ON, Canada

## Abstract

**Introduction:**

Diarrhea and pneumonia are the two leading causes of mortality in children under five. Improvements have occurred over the past two decades but the progress is slow to meet the MDG-4.

**Methods:**

We conducted a systematic review of the randomized controlled trials, quasi-experimental and observational studies to estimate the effect of community based interventions including community case management on the coverage of various commodities and on mortality due to diarrhea and pneumonia. We used a standardized abstraction and grading format and performed meta-analyses for all the relevant outcomes. The estimated effect of community based interventions was determined by applying the standard Child Health Epidemiology Reference Group (CHERG) rules.

**Results:**

We included twenty four studies in this review. Community based interventions led to significant rise in care seeking behaviors with 13% and 9% increase in care seeking for pneumonia and diarrhea respectively. These interventions were associated with 160% increase in the use of ORS and 80% increase in the use of zinc for diarrhea. There was a 75% decline in the unnecessary use of antibiotics for diarrhea and a 40% decrease in treatment failure rates for pneumonia. Community case management for diarrhea and pneumonia is associated with a 32% reduction in pneumonia specific mortality, while the evidence on diarrhea related mortality is weak.

**Conclusion:**

Community based interventions have the potential to scale up care seeking and the use of essential commodities and significantly decrease morbidity and mortality burden due to diarrhea and pneumonia in children under the age of five years.

## Introduction

Approximately 6.9 million deaths of children under five years occurred in 2011 due to preventable and treatable causes [1]. Diarrhea and pneumonia were the two leading causes of mortality accounting for about 29% of the total burden [2] and more than 95% of this burden is shared by the 75 Countdown countries. The incidence of diarrhea has declined from 3.4 episodes/child year in 1990 to 2.9 episodes/child year in 2010 [3]. Improvements have been observed, but over a greater span of time [4] and this reduction has unfortunately not been enough to bring us within the reach of the MDG 4, which is to achieve two thirds reduction in the mortality of children under the age of five globally, by the year 2015 [4].

One of the causes for the delay in meeting these targets is the poor access to health facilities and paucity of trained human resources in primary health setups. This deprives the poorest of the populations of simple and effective interventions, like Oral Rehydration Solution (ORS), which has huge potential to save lives. Measures are required to increase awareness among the masses and one such way, is to provide these services through community platforms like home visitation and community based delivery mechanisms. Community Health Workers (CHWs) have been involved in this respect by a few countries especially targeting pneumonia and diarrhea. There has been an increase in programs that train CHWs in poor resource settings to deliver life-saving interventions to children at scale, thus validating the quality of the care they deliver [5,6]. CHWs in these programs have been assigned the task to educate mothers, diagnose and treat by prescribing antimicrobials and referring complicated cases to first level referral facilities or district hospital. Roesin et al [7] provides evidence that a community based program involving health education by CHWs increased care-seeking for pneumonia in Indonesia and a study from Thailand provides similar evidence [8]. An evaluation of a community-based programme in Matlab, Bangladesh, provides evidence that active case detection and referral to facilities by CHWs can have a beneficial effect on pneumonia mortality [9]. Proper program planning at the initial stage, involving careful hiring, training, intensive field supervision, support and incentives in some cases are the basis of success. In Bangladesh, a study found 87% agreement between treatments recommended by CHWs and by a study physician for children with suspected pneumonia. Training of these CHWs is the most vital aspect in correct diagnosis and management as shown in Bangladesh BRAC-supported CHWs ‘basic’ training program which reported that the more the exposure to training, the better was the diagnosis and management of pneumonia [10,11].

Few children in the developing world receive appropriate treatment for diarrhea and pneumonia, even the simple and cost effective interventions like continued feeding, has a coverage of just 39% and this is even low for ORS [2]. The last decade has been stagnant in terms of the improvement in coverage of essential commodities like ORS and zinc for diarrhea and antibiotics for pneumonia. Recent surveys indicate that worldwide, 78% of children under-five with symptoms of pneumonia are taken to an appropriate provider; in low-income countries, this coverage is 43%. Antibiotics have an essential role in reducing deaths due to pneumonia and in low-income countries, less than one-third (29%) of under-five children with symptoms of pneumonia receive antibiotics [2].

We in this review have estimated the effect of these Community Based Interventions (CBIs) on the care seeking behavior coverage and uptake of essential commodities for diarrhea and pneumonia: ORS, zinc therapy for diarrhea and antibiotics for pneumonia. We have also assessed the impact on negative practices like prescribing unnecessary antibiotics for diarrhea. Previous review by Theodoratou et al [12] estimated that Community Case Management (CCM) of pneumonia could result in a 70% reduction in mortality from pneumonia in 0–5-year-old children. We in this review have updated the previous estimate and also estimated the effect of case management on diarrhea mortality. We have reviewed the available literature and evaluated the quality of included studies according to the Child Health Epidemiology Reference Group (CHERG) adaptation of Grading of Recommendations, Assessments, Development and Education (GRADE) criteria [13].

## Methods

We systematically reviewed all literature published up to Nov 2012 to identify studies describing the effectiveness of CBIs on diarrhea and pneumonia in children less than or equal to 5 years. Following CHERG Systematic Review Guidelines, we searched PubMed, Cochrane Libraries, Embase, and WHO Regional Databases to identify the studies. We included randomized controlled trials, quasi-experimental and observational studies. Additional studies were identified by hand searching references from included studies. No language or date restrictions were applied in the search.

### Inclusion criteria

Studies were included if they reported the effect of CBIs on care seeking behaviors and uptake of essential commodities or case management on morbidity and mortality associated with diarrhea and pneumonia. Studies were included only if a clear case definition of diarrhea and pneumonia were used. The primary outcome measures for CBI were: care seeking rates, use of ORS and zinc for diarrhea, antibiotics use and treatment failure rates for diarrhea and pneumonia and for case management: incidence of moderate or severe episodes of acute lower respiratory infection (ALRI), diarrhea-specific mortality, pneumonia-specific mortality and all-cause mortality.

### Abstraction, analysis and summary measure

For the studies that met the final inclusion criteria, we abstracted data describing study identifiers and context, study design and limitations, intervention specifics and outcome effects into a standardized abstraction form as detailed in the CHERG Systematic Review Guidelines. Each study was assessed and graded according to the CHERG adaptation of the GRADE technique.

### Quantitative data synthesis

We conducted meta-analyses for individual studies and pooled statistics was reported as the relative risk (RR) between the experimental and control groups with 95% confidence intervals (CI). Mantel–Haenszel pooled RR and corresponding 95% CI were reported or the DerSimonian–Laird pooled RR and corresponding 95% CI where there was an unexplained heterogeneity. All analyses were conducted using the software Review Manager 5.1. Heterogeneity was quantified by Chi^2^ and I^2^, which can be interpreted as the percentage of the total variation between studies that is attributable to heterogeneity rather than to chance, a low p-value (less than 0.1) or a large chi-squared statistic relative to its degree of freedom and I^2^ values greater than 50% were taken as substantial and high heterogeneity. In situations of high heterogeneity, causes were explored by sensitivity analysis and random effect models were used. A subgroup analysis was also performed based on the different age groups. We summarized the evidence by outcome, including qualitative assessments of study quality and quantitative measures, according to the standard guidelines. A grade of “high”, “moderate”, “low” and “very low” was used for grading the overall evidence indicating the strength of an effect on specific health outcome according to the CHERG Rules for Evidence Review [13].

## Results

**Figure 1:**
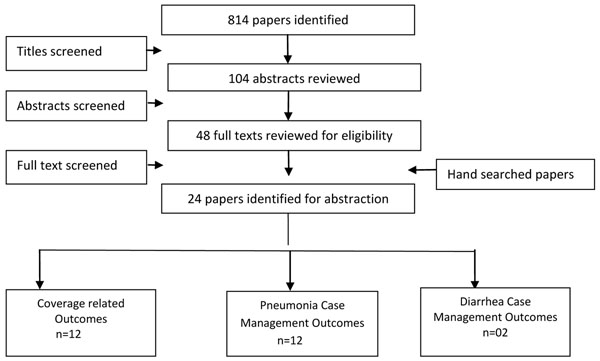
Search strategy flow diagram

We identified 814 papers from search in all databases. After the initial title and abstract screening, 48 full texts were reviewed to identify papers which met the inclusion criteria and had outcomes of our interest. Twenty four papers [14-37] were finally selected for abstraction and analysis (Figure 1). Two studies [15,26] did not report enough data to be included in the meta-analyses. We also included two unpublished trials (Habib et al and Soofi et al) after the author’s permission; both of these studies were conducted in Pakistan. All included studies were conducted in Asia or Africa.

### Coverage impacts

**Figure 2:**
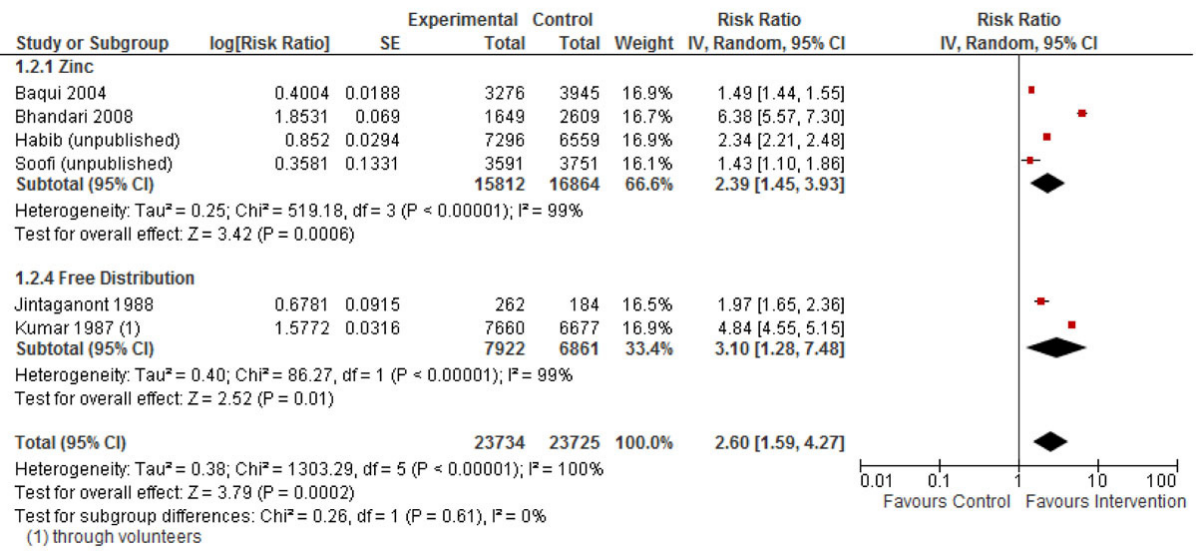
Forest plot of effect of community based interventions on the use of ORS for diarrhea

In Table 1, we report the quality assessment of studies by outcomes of coverage indicators. The community based interventions significantly improved care seeking for diarrhea and pneumonia by 9% (RR: 1.09, 95% CI: 1.06, 1.11) and 13% (RR: 1.13, 95% CI: 1.08, 1.18) respectively. The use of ORS increased significantly by 160% (RR: 2.60, 95% CI: 1.59, 4.27) (Figure 2) and these CBIs resulted in a 29 folds rise in the use of zinc (RR: 29.79, 95% CI: 12.33, 71.97) in the management of diarrhea. The use of antibiotics for diarrhea reduced significantly by 75% (RR: 0.25, 95% CI: 0.12, 0.51), while there was an insignificant impact on use of antibiotics for pneumonia (RR: 1.13, 95%CI: 0.99, 1.30), although one study was evaluated for this outcome. The rate of treatment failure for pneumonia also reduced significantly by 40% (RR: 0.60, 95% CI: 0.51, 0.70).

**Table 1:** Effect of community based interventions on the coverage of commodities/services for diarrhea and pneumonia

	Quality Assessment					Summary of Findings		
				Directness		No of events		

No of studies	Design	Limitations	Consistency	Generalizability to population of interest	Generalizability to intervention of interest	Intervention	Control	Relative Risk (95% CI)

*Care seeking rates for Pneumonia: moderate outcome specific quality of evidence*								

Two	RCT/Quasi	No significant heterogeneity so a fixed effect model used	Both studies suggest benefit	One study from Asia and one from Africa	WHO case management by local health workers	344	327	1.13 [1.08, 1.18]

*Care seeking rates for Diarrhea: moderate outcome specific quality of evidence*								

Four	CRCT	Significant heterogeneity so a random effect model used	Two studies show benefit	All studies from South Asia	Promotion of use of ORS and zinc by CHWs	3562	4691	1.09 [1.06, 1.12]

*ORS use for the management of diarrhea: moderate/low outcome specific quality of evidence*								

Six	RCT/Quasi	Significant heterogeneity so a random effect model used	All the studies suggest benefit	All studies are from Asia	All the studies had community education while four studies had combined intervention of promotion and zinc therapy and two had free distribution of ORS	10446	3990	2.60 [1.59, 4.27]

Two	Before/After	No major limitation	All the studies suggest benefit	Both studies were from Africa	One study had combined intervention of promotion of zinc therapy	143	86	1.75 [1.48, 2.07]

*Use of zinc for the management of diarrhea: moderate outcome specific quality of evidence*								

Four	cRCT	Significant heterogeneity across studies so a random effect model used	All Studies suggest benefit	All studies from South Asia	CHWs provided education and promoted use of ORS and zinc	5554	14	29.79 [12.33, 71.97]

Antibiotic Use for Diarrhea: moderate outcome specific quality of evidence								

Four	cRCT	Significant heterogeneity across studies so a random effect model used	All studies suggested a decline in the use of antibiotics	All studies from South Asia	CHWs provided education and promoted use of ORS and zinc	639	3083	0.25 [0.12, 0.51]
One	Before/After		Decline in the use of antibiotics	Study conducted in Mali	CHWs provided education and promoted use of ORS and zinc	104	130	0.83 [0.69, 0.99]

Antibiotic Use for Pneumonia: moderate outcome specific quality of evidence								

One	Quasi			Study Conducted in Uganda	WHO case management by local health workers	187	319	1.13 [0.99, 1.30]

Treatment Failure Rates for ARI/Pneumonia: moderate outcome specific quality of evidence								

Two	cRCT	No significant heterogeneity so a fixed effect model used	Both studies suggest benefit	One study from Asia and one from Africa	WHO case management by local health workers	228	314	0.60 [0.51, 0.70]

### Case management for pneumonia

**Figure 3:**
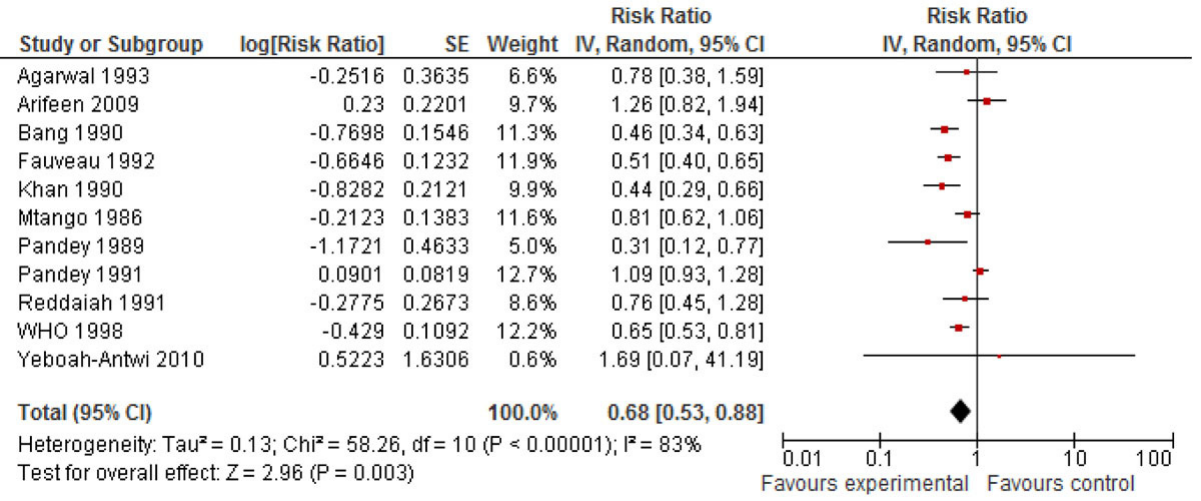
Forest plot of effect of community case management on pneumonia mortality

In Table 2, we report the quality assessment of studies by outcomes of pneumonia. The estimated impact of CCM on pneumonia-specific mortality in 0-1 month age group was a 42% reduction (RR: 0.58, 95%CI: 0.44, 0.77), this result was based on four concurrent studies, a 42% (RR: 0.58, 95%CI: 0.50, 0.67) reduction was also estimated for the 0-1 year age group in which nine concurrent and before/after studies were evaluated. In the 1-4 year age group, there was an insignificant 49% (RR: 0.51, 95%CI: 0.24, 1.07) reduction based on two before/after studies, while there was a 32% (RR: 0.68, 95%CI: 0.53, 0.88) reduction in ALRI mortality based on eleven concurrent and before/after studies (Figure 3).

**Table 2:** Quality assessment of studies of community case management for the treatment of pneumonia

	Quality Assessment					Summary of Findings		
				Directness		No of events		

No of studies	Design	Limitations	Consistency	Generalizability to population of interest	Generalizability to intervention of interest	Intervention	Control	Relative Risk (95% CI)

*ALRI mortality 0–1 months: moderate outcome specific quality of evidence*								

Four	Concurrent	No Major	3 of 4 studies show benefit	Africa and Asia	3 of 4 studies WHO case management by local health workers	384	686	0.58 (0.44–0.77)

*ALRI specific mortality 0–1 year: moderate outcome specific quality of evidence*								

Six	Concurrent	No major limitations	Heterogeneity from meta-analysis, All studies show benefit	Africa and Asia	4 of 6 studies WHO case management	916	1510	0.59 (0.46–0.75)
Two	Before/After	High ALRI incidence	Heterogeneity from meta-analysis, All studies show benefit	Asia	1 of 2 studies WHO case management	7	34	0.36 (0.16–0.82)
Seven	Concurrent; before/ after	See Above	Heterogeneity from meta-analysis, All studies show benefit	Africa and Asia	See Above	917	1522	0.57 (0.44–0.75)
Nine	Concurrent; before/ after	See Above	Heterogeneity from meta-analysis, All studies show benefit	Africa and Asia	See Above	938	1569	0.58 (0.50– 0.67)

*ALRI-specific mortality 1–4 years: low outcome specific quality of evidence*								

Two	Before/After	High ALRI incidence	Both studies show benefit	Asia	1 of 2 studies WHO case management	10	24	0.51 (0.24–1.07)

ALRI-specific mortality 0-4 years: moderate outcome specific quality of evidence								

Eight	Concurrent	No major limitation	Five of eight studies show benefit	Africa and Asia	6 of 8 studies WHO case management	705	948	0.68 [0.53, 0.86]
Six	Before/After	No major limitation	Four of six studies show benefit	Africa and Asia	3 of 5 studies WHO case management	220	271	0.77 [0.54, 1.08]
Ten	Concurrent; before/ after	See Above	Eight of ten studies show benefit	Africa and Asia	See Above	724	986	0.67 [0.51, 0.88]
Eleven	Concurrent; before/ after	See Above	Nine of eleven studies show benefit	Africa and Asia	See Above	744	1032	0.68 [0.53, 0.88]

All cause mortality 0–1months: moderate outcome specific quality of evidence								

Five	Concurrent	No major limitation	All studies show benefit	Africa and Asia	4 of 5 studies WHO case management	925	957	0.73 (0.65– 0.82)

All-cause mortality 0–1 year: moderate outcome specific quality of evidence								

Six	Concurrent	No Major limitation	All studies show benefit	Africa and Asia	4 of 6 studies WHO case management	2095	2487	0.78 (0.71– 0.85)
Two	Before/After	High ALRI incidence	All studies show benefit	Asia only	1 of 2 studies WHO case management	41	100	0.60 (0.42–0.85)
Seven	Concurrent; before/ after	See above	All studies show benefit	Africa and Asia	See above	2114	2524	0.77 (0.70– 0.85)
Nine	Concurrent; before/ after	See Above	All studies show benefit	Africa and Asia	See above	2230	2703	0.79 (0.72–0.86)

All-cause mortality 1–4 years: low outcome specific quality of evidence								

Two	Before/After	High ALRI incidence	Both studies show benefit	Only Asia	1 of 2 studies WHO case management	43	82	0.49 (0.34–0.70)

All-cause mortality 0–4 years: moderate outcome specific quality of evidence								

Nine	Concurrent	No major limitation	Six studies show benefit	Africa and Asia	6 of 9 studies WHO case management	3115	4180	0.83 [0.73, 0.95]
Six	Before/After	No major limitation	Four studies show benefit	Africa and Asia	3 of 6 studies WHO case management	1141	1063	0.90 [0.64, 1.26]
Eleven	Concurrent; before/ after	No major limitation	See Above	Africa and Asia	See Above	3113	4401	0.80 [0.77, 0.83]
Twelve	Concurrent; before/ after	No major limitation	See Above	Africa and Asia	See Above	3214	4473	0.80 [0.77, 0.83]

The estimated reduction in all-cause mortality in 0-1 month age group was 27% (RR: 0.73, 95% CI: 0.65, 0.82), this result was based on five concurrent studies. A 21% (RR: 0.79, 95%CI: 0.72, 0.86) reduction was estimated for the 0-1 year age group in which nine concurrent and before/after studies were evaluated. In the 1-4 years age group, there was a 51% (RR: 0.49, 95%CI: 0.34, 0.76) reduction based on two before/after studies, while there was a 20% (RR: 0.80, 95%CI: 0.77, 0.83) reduction in all-cause mortality based on twelve concurrent and before/after studies.

### Case management for diarrhea

**Figure 4:**
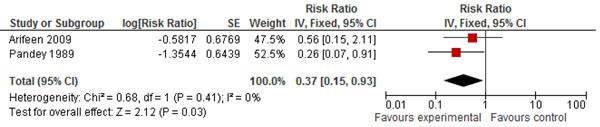
Forest plot of effect of community case management on diarrhea mortality

In Table 3, we report the quality assessment of studies by outcomes of diarrhea. There were two studies identified which had evaluated the effect of CCM on diarrhea. The estimated impact on diarrhea specific mortality in the 0-1 year age group was 92% (RR: 0.08, 95%CI: 0.00, 0.87) reduction, this result was based on only one before/after study. In the 1-4 years age group, only one before/after study was evaluated and it showed an increase in the diarrhea specific mortality (RR: 2.98, 95%CI: 0.31, 28.63). While in the 0-4 years age group, there was a 63% (RR: 0.37, 95%CI: 0.15, 0.93) reduction based on two concurrent and before/after studies (Figure 4). The effect on all-cause mortality was based on two concurrent and before/after studies and it showed an insignificant 6% (RR: 0.94, 95%CI: 0.78, 1.12) reduction.

**Table 3:** Quality assessment of studies of community case management for the treatment of diarrhea

	Quality Assessment					Summary of Findings		
				Directness		No of events		

No of studies	Design	Limitations	Consistency	Generalizability to population of interest	Generalizability to intervention of interest	Intervention	Control	Relative Risk (95% CI)

*Diarrhea Specific mortality 0–1 years: low outcome specific quality of evidence*								

One	Before/ After	No major limitation		Asia Only	WHO case management by local health workers	0	11	0.05 [0.00, 0.87]

Diarrhea Specific mortality 1-4 years: low outcome specific quality of evidence								

One	Before/ After	No major limitation		Asia Only	WHO case management by local health workers	3	1	2.98 [0.31, 28.63]

*Diarrhea Specific mortality 0-4 years: moderate outcome specific quality of evidence*								

One	Concurrent	No major limitation		Asia Only	WHO case management by local health workers	3	8	0.56 [0.15, 2.11]
Two	Before/After	No major limitation	Both studies show benefit	Asia Only	WHO case management by local health workers	6	19	0.32 [0.13, 0.80]
Two	Concurrent: Before/After	No major limitation	Both studies show benefit	Asia Only	WHO case management by local health workers	6	20	0.37 [0.15, 0.93]

All-cause mortality 0–4 years: moderate outcome specific quality of evidence								

One	Concurrent	No major limitation		Asia Only	WHO case management by local health workers	157	221	1.06 [0.86, 1.30]
Two	Before/After	No major limitation	One shows benefit	Asia Only	WHO case management by local health workers	194	208	0.80 [0.47, 1.35]
Two	Concurrent; Before/After	No major limitation	One shows benefit	Asia Only	WHO case management by local health workers	194	285	0.94 [0.78, 1.12]

### Recommendation for the LiST model

**Figure 5:**
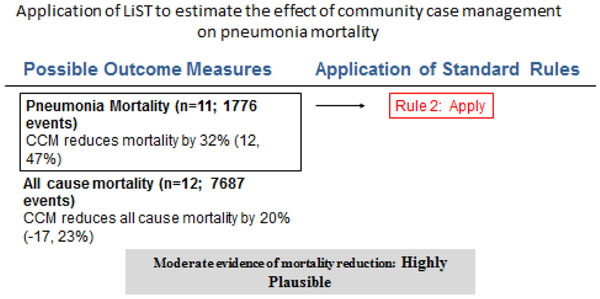
Application of standardized rules for choice of final outcome to estimate effect of community case management on pneumonia mortality

Of the outcomes assessed for the effect of CCM on pneumonia and diarrhea in children, we applied the CHERG rules for evidence review. For pneumonia, we applied rule number 2 of effect on cause-specific mortality, to propose that CCM is associated with a 32% (12%, 47%) reduction in pneumonia-specific mortality (Figure 5). This estimate is based on analysis of eleven concurrent and before/after studies with 1776 events (Figure 5). For diarrhea the evidence was weak, as there were less than 50 events in cause-specific mortality and the estimate was insignificant for all-cause mortality.

For coverage impacts we propose a 160% (59%, 427%) increase in the use of ORS, this estimate was based on six concurrent studies with 14436 events and a 75% (49%, 88%) reduction in the use of antibiotics for diarrhea based on four concurrent studies with 3722 events. We also propose a 80% increase in the use of zinc for diarrhea; this estimate is based on the percentage difference in the use of zinc amongst the intervention and control groups. For pneumonia related outcomes, we propose 40% (30%, 49%) reduction in the treatment failure rates based on two concurrent studies with 542 events.

## Discussion

Community based interventions increase the uptake of services in resource deprived populations with diarrhea and pneumonia. This review further strengthens the evidence shown by previous reviews on the effectiveness of case management.

The CBIs show a significant increase in the uptake of the WHO recommended treatment modalities for diarrhea (ORS and zinc) and at the same time reducing unnecessary use of antibiotics for diarrhea by 75%. For pneumonia, these interventions increased the care seeking behavior by 13% and the treatment failure rates also reduced by 40%. There was evidence of heterogeneity across the studies in our meta-analyses; however, overall there is consistency in the direction of effect. Our analysis also shows the benefit of case management on pneumonia treatment, as it was associated with a 32% reduction in pneumonia related mortality and a positive trend was noted in the management of diarrhea.

The models of care were not consistent across studies, with variations in training, diagnostic guidelines and antibiotic prescriptions. But most of the studies have shown significant effects in terms of coverage, access and curative outcomes. This approach also allows the care to be provided near to home, thus averts the need for transportation and saves productive time for the child’s care provider. CBI primarily operates through increased access and consequently increasing care-seeking behavior. This approach also highlights the importance of mother’s education for the long term sustainability and success of this approach. Although the community should be educated, as more awareness amongst care providers would lead to more care seeking, but instant effects from the case management approach should not be expected. Apart from improved access and coverage, these CBIs also lead to improved quality of services and were successful in the early diagnosis and treatment. These programs were also well accepted by the community.

While CHWs can be trained in a minimum of six days; a lot of resources and infrastructure is required for this approach to work at its optimum. The staff and essential commodities at these facilities should be adequate as shortage could discourage care providers from visiting the facility. A study [38] estimated that access to case management is much worse than officially estimated if contribution of physical barriers, staff availability and stock outs were accounted for. It showed that less than 50% of the population had geographic access (i.e., lived within 10 km of a facility), and less than 20% had effective access.

Training of CHWs is the most important component in the correct diagnosis and management. However, correct treatment was poor when there was a long gap (one year) between the training and the follow up [39], so regular refresher trainings are recommended, which would help keep the standards consistent. These CHWs in majority of the programs were not linked to the formal health system of the country and were expected to work as volunteers, which were noticed as a drawback so incentives need to be added to these programs to gain additional grounds. In Pakistan, Lady Health Workers (LHWs) are considered civil servants and given one-year contracts, hence retention rate is high in these areas. In the first three years of the program, an average of 5.4 per cent of LHWs dropped out per year while in recent years, dropouts have averaged less than 1 per cent per year [40].

Despite of these initial gains, a lot more and sustained effort is required to ensure that children receive appropriate treatment for pneumonia and diarrhea on a larger scale. Barriers such as cultural, economic, and social constraints need to be addressed for the universal uptake. Countries accounting for nearly a quarter of annual global pneumonia mortality, many with low coverage of facility-based treatment do not implement CCM of childhood illnesses. Where CCM is implemented, it often occurs on a limited scale or in pilot projects, commonly supported by international agencies and donors.

Standardization is also needed, in defining case management of childhood illnesses. The global public health community needs an operational definition of this strategy to better describe, monitor and evaluate CCM programs. There is a need for greater accountability in maternal, newborn, and child health programs and this poses new challenges to governments on how to conduct regular assessments of the quality of health services delivered at community level [41].

This approach can reach its full potential if other factors related to child mortality; vaccination, hand washing, hygiene, are also taken care of. While our findings are promising, we suggest areas for further operational research to strengthen CBIs program learning and functioning.

## Authors' contributions

Dr. ZAB was responsible for designing the review and co-ordinating the review. JKD, ZL and RAS were responsible for: data collection, screening the search results, screening retrieved papers against inclusion criteria, appraising quality of papers, abstracting data from papers, entering data into RevMan, analysis and interpretation of data and writing the review. ZAB and JKD critically reviewed and modified the manuscript.

## Competing interests

The authors declare they have no conflict of interest.

## Declarations

The publication costs for this supplement were funded by a grant from the Bill & Melinda Gates Foundation to the US Fund for UNICEF (grant 43386 to "Promote evidence-based decision making in designing maternal, neonatal, and child health interventions in low- and middle-income countries”). The Supplement Editor is the principal investigator and lead in the development of the Lives Saved Tool (LiST), supported by grant 43386. He declares that he has no competing interests.

This article has been published as part of *BMC Public Health* Volume 13 Supplement 3, 2013: The Lives Saved Tool in 2013: new capabilities and applications. The full contents of the supplement are available online at http://www.biomedcentral.com/bmcpublichealth/supplements/13/S3.
